# “I Think the Mental Part Is the Biggest Factor”: An Exploratory Qualitative Study of COVID-19 and Its Negative Effects on Indigenous Women in Toronto, Canada

**DOI:** 10.3389/fsoc.2022.790397

**Published:** 2022-05-02

**Authors:** Jerry Flores, Kristen Emory, Xuan Santos, Angela Mashford-Pringle, Kati Barahona-Lopez, Keston Bozinovic, Jennifer Adams, Coco Chen, Yandy Zuo, Diana Nguyen

**Affiliations:** ^1^Department of Sociology, University of Toronto, Mississauga, ON, Canada; ^2^School of Public Health, San Diego State University, San Diego, CA, United States; ^3^Department of Sociology and Criminology and Justice Studies, California State University San Marcos, San Marcos, TX, United States; ^4^Dalla Lana School of Public Health, University of Toronto, Toronto, ON, Canada; ^5^Department of Sociology, University of Wisconsin-Eau Claire, Eau Claire, WI, United States

**Keywords:** COVID-19 pandemic, Indigenous, qualitative study, urban, Toronto (Canada)

## Abstract

This article explores the unique and understudied experiences of Indigenous women living in Toronto, Canada during the first year of the COVID-19 pandemic. The purpose of this study is to better document the impacts of COVID-19 on the mental health and wellbeing of Indigenous women in Toronto, Canada to better understand unmet needs, as well as lay the groundwork for more targeted research and potential interventions based on these needs. Using in-depth semi-structured interviews with thirteen Indigenous women, we shed light on the negative effects this pandemic has had on this population. We find that COVID-19 has negatively affected people's mental health, substance use and access to health services. This research speaks to the growing body of work that discusses the harmful effects of COVID-19 generally and how this pandemic has specifically affected Indigenous peoples.

## Introduction

The COVID-19 pandemic has had widespread negative effects on communities across the world (Levy Economics Institute, 2020). Additionally, COVID-19 has exacerbated existing inequalities (Hu, 2020). This is especially true for the most marginalized peoples (Luna, 2020). Currently, there is little research related to how COVID-19 has negatively affected these communities. Even less research looks at how COVID-19 has negatively affected Indigenous peoples in various ways (Howard-Bobiwash et al., 2021). Scientific evidence regarding the impact of COVID-19 on people's lives is limited and still emerging; the evidence that does exist often does not include an in-depth assessment of the impact on Indigenous populations. Lack of inclusion of Indigenous populations in scientific inquiry is particularly concerning as without documentation health equity concerns can go unnoticed by public health professionals as well as governmental offices in charge of funding population health work. There is consistent emerging evidence that in addition to the physical symptoms of COVID-19, the pandemic is also negatively impacting the mental health of populations across the globe (Dong and Bouey, 2020; Fiorillo and Gorwood, 2020; Passos et al., 2020; Sheridan Rains et al., 2021). This negative impact may be exacerbated for historically marginalized populations, making it all the more vital that their perspectives are heard and addressed. They study of women (women of color in particular) is especially important given that research demonstrate that they have disproportionately suffered from mental and physical health issues compared to men since the start of this pandemic (Gomez-Aguinaga et al., 2021; Luo and Sato, 2021; Ornelas et al., 2021; Priebe Rocha et al., 2021).

The current study is an attempt to bridge this gap in the literature and provide a foundation for future scientific inquiry. Using in-depth semi-structured interviews with 13 Indigenous women in the Greater Toronto Area, we address the following questions in this paper: how did the first year of the COVID-19 pandemic affected the mental health of the 13 Indigenous women participants in Toronto, Canada? Furthermore, how has it negatively affected our participants' mental health and patterns of substance, alcohol, and tobacco use? The following paper shows how COVID-19 has adversely affected 13 Indigenous women. In the following sections, we review work on mental health and BIPOC (Black Indigenous People of Color) populations, mental health, and its effects on substance abuse among Indigenous peoples. We then discussed the methodological approach used in this paper and our main findings, which we divided into three small sections. We follow this with a discussion of our results and their implications for Indigenous peoples and future work related to COVID-19 and this community.

### Mental Health and Black, Indigenous, and People of Color Communities

To better understand the impact of COVID-19 on the mental health of Indigenous women in Canada, it is important to understand the larger social context of mental health and related health equity concerns among diverse and often historically marginalized communities. Black, Indigenous, and People of Color (BIPOC) populations in the aggregate often experience disparate mental, physical, social, and economic risk factors and health outcomes, often due to structural and systemic inequity (Gee and Ford, 2011). Mental health is a particular concern as it impacts and is impacted by so many aspects of the human health and wellbeing experience.

Adverse mental health in the general global population was notably elevated during the COVID-19 pandemic (Czeisler et al., 2020). Subsequently, many people began or increased substance use to cope with economic stress, loneliness, and anxiety surrounding the virus in conjunction with pre-existing daily stressors (Czeisler et al., 2020). Communities that were previously at risk before the pandemic became particularly vulnerable during the pandemic due to social and health inequities (Abrams and Szefler, 2020). There was a notable increase in usage of alcohol (Pollard et al., 2020), nicotine and tobacco (Giovenco et al., 2021), opioids (Niles et al., 2021), and marijuana, along with other psychoactive substances (Borgonhi et al., 2021).

For communities of color, especially communities that are historically marginalized and colonized like the Black (Millett et al., 2020), Latinx (Macias Gil et al., 2020), and Indigenous (BIPOC) communities (Yellow Horse et al., 2020), their mental health was negatively impacted by intergenerational trauma, ongoing police violence (DeVylder et al., 2020), oppression related to poverty and racism, and the devastating burden of COVID-19 in these communities. For example, Black respondents reported increased rates of substance use and suicidal ideation (Czeisler et al., 2020). Hispanic/Latinx respondents reported a higher prevalence of anxiety disorder and depressive disorder symptoms, COVID-19 related trauma- and stressor-related disorder (TSRD), increased substance use, and suicidal ideation (Czeisler et al., 2020).

Indigenous communities in the US, American Indians/Alaska Natives (AI/AN), have had disproportionately higher rates of substance abuse (Dickerson et al., 2011; Statistics Canada, 2011; Wolfe et al., 2018; Gonzalez et al., 2019), which likely increased during COVID-19 in conjunction with other adverse mental health conditions. In addition, LGBTQ+ populations face the risk of worse COVID-19 health outcomes due to higher rates of comorbidities, working in affected industries as essential workers, being more likely to be low-income/affected by poverty, experiencing stigma or discrimination due to gender identity or sexual orientation, and lack of access to insurance and healthcare (Dawson and Golijani-Moghaddam, 2020). These inequities may also put LGBTQ+ populations at increased risk of stress and adverse mental health (Dawson and Golijani-Moghaddam, 2020).

As a whole, the impact of the COVID-19 pandemic has disproportionately affected BIPOC communities (Cheung, 2020). While they are systemically underrepresented in both research and government data collection, there is still clear evidence that racism is a risk factor for COVID-19 mortality (Wallis, 2020). Canadian health researchers have confirmed that structural inequalities related to race and gender, including healthcare, labor, and community affluence, influence the disproportionate impact of COVID-19 (Slaughter, 2020). Three key social issues have been identified as contributors to the interrelationship between racism and COVID-19 in Canada, leading to inequitable health outcomes in BIPOC communities: the healthcare system, occupation, and living conditions within the home and the community (Learning Network, 2021). Bias toward marginalized groups, including Indigenous populations, has been documented as a recurring problem in Canada's healthcare system, which results in lower quality of care, leaving communities vulnerable to potential cases of COVID-19 (Skosireva et al., 2014; Morris et al., 2019; Wylie and McConkey, 2019).

These contributing factors are further stratified among gender, especially since BIPOC women are overrepresented in jobs with a higher risk of exposure to COVID-19 while generally being in lower-paying positions (Learning Network, 2021). These systemic contributors to health inequity combined with higher rates of unemployment, incarceration, and substance use in BIPOC communities compound the harmful effects of COVID-19, often leading to more severe morbidity outcomes and higher rates of mortality (Dickerson et al., 2011). Further impacting the health outcomes related to COVID-19, it has been well documented that factors like low socioeconomic status, limited access to resources, and stigmatization can affect the likelihood that a person will experience poor mental health or engage in substance use.

### Mental Health and Corresponding Substance Use Among Indigenous Populations

The historical trauma of being oppressed by the Canadian government and being forced to civilize, assimilate, and eliminate their cultures has been detrimental to the mental health of Indigenous people (Boksa et al., 2015). Social determinants such as social exclusion, discrimination, poverty and unemployment have always played a significant role in the mental health challenges faced by the Indigenous population (Boksa et al., 2015). These mental health challenges worsened due to the COVID-19 pandemic. For example, Statistics Canada conducted a crowdsourcing data collection consisting of 1,400 Indigenous participants. The crowdsourced data showed that six in ten Indigenous participants reported experiencing a decline in their mental health since the onset of physical distancing due to COVID-19 (Statistics Canada, 2020). Compared to Indigenous men, Indigenous women reported experiencing higher stress and anxiety due to “multiple caregiving burdens, risks of gender-based violence, and economic vulnerabilities” (Statistics Canada, 2020, p. 4). Among the participants, 46% of Indigenous women and 32% of Indigenous men described their days during COVID-19 as being “quite a bit stressful” or “extremely stressful” and reported having symptoms of anxiety (Statistics Canada, 2020). Overall, higher proportions of Indigenous participants reported having poor mental health and higher stress and anxiety than non-Indigenous people. The COVID-19 pandemic exacerbated this dynamic.

## Methods

In March of 2020 the COVID-19 pandemic was in full swing. During this time the University of Toronto put a halt to all in person interviews and data collection. This meant that the ethnographic research we were conducting with Indigenous women in Toronto had to pause. During this time we began to wonder about the challenges Indigenous women faced during the pandemic? Given this interest and a small grant provided by the Sociology department at the University of Toronto Mississauga we decided to ask Indigenous women about their experiences during COVID-19. We decided to conduct follow-up interviews with individuals included in our larger study on Missing and Murdered Indigenous women in Toronto. In other words, we had established relationships with our participants and reached out again after the start of the COVID-19 pandemic. These relationships made including them in this study less challenging.^1^ This is especially important given the negative history between researchers and Indigenous peoples in Canada and across the world (Wilson, 2008). When conducting research involving Indigenous people in Canada, it is crucial to be aware and cognizant of the traumatic history and ongoing harm and exploitation researchers can inflict on this community. We attempted to not contribute to this negative history during this research. Additionally, while we focused on women we already knew, we were also open to speaking with people other individuals who are respondents suggested we include.

During a period of 6 months, we conducted a series of semi-structured qualitative interviews with Indigenous women living in Canada.^2^ We initially reached out via email or text message to gauge the interest of potential participants. Thirteen individuals returned our correspondence. We then conducted a series of hour-long interviews via phone and video call which took place during one of the various lockdowns that occurred in the city. Most of the interviews took place during the day and we used handheld records to document our conversations. We allowed respondents to choose their preferred method of communication for these interviews. During these calls, two researchers were present. One asked interview question, and another took field notes. After conducting these interviews, we mailed all participants a $50 gift card. Additionally, we informed them that they could change their mind about participating in this study and still keep their compensation.

All the women in this study self-identified as Indigenous from various nations in Canada. They ranged in age from 22 to 60. Most were experiencing economic hardship during the time, which was largely related to COVID-19. All research in this study was approved by the ethics board at our respective universities and received consent/assent from the interviewees. Fieldnotes and interviews were transcribed verbatim by authors. The authors then used *Dedoose*, a qualitative data analysis software package, to code these documents. During the coding process we looked for patterns or recurring themes in our interviews in notes. We then grouped these patterns into larger “themes.” Themes that appeared the most became the foundation of the findings we describe later in the paper. This method of analyzing, organizing and coding ethnographic data follows the process described in Emerson et al. (1995). Apart from what we share in this paper women's problems with finances, home schooling children and themselves as well as access to transportation also emerged as a prominent theme and will form the basis of a separate publication. We included a Table 1 with participants demographic information at the end of this document.^2^ This is information our participants shared during the interviews.

**Table 1:** Participants information.

**Name**	**Ethnicity/nation**	**Age**	**Socioeconomic status**	**Education**	**Number of children**
Susan	Algonquin	61	N/A	College graduate	0
Ciara	N/A	36	Middle	College graduate	4
Jenny	White Fish River First Nation	50	Low	N/A	2
Lina	N/A	54	Low	N/A	0
Gina	N/A	23	Low	University graduate	2
Alice	Ojibway	35	Low	Partial high school	6
Jennifer	Métis	40	Middle	University graduate	3
Patty	N/A	27	N/A	Partial high school	0
Laura	Six Nations	57	N/A	College graduate	3
Erin	N/A	60	Low	Partial high school	3
Robin	N/A	N/A	Low	High school graduate	1
Lana	First Nations	N/A	Low	Partial university	2
Jill	Mi'kmaq	25	Upper	University graduate	0

### Reflexivity

Reflexivity allows scholars to connect their experiences of oppression and privilege to their research activities (Rios, 2011; Flores, 2016; Flores et al., 2019). It reveals the tenuous lines involved during empirical research that include relations between researcher and self, researcher and participants, and researchers and their readers/audiences (Doucet, 2008). Both authors share a background in ethnographic training and a commitment to social justice. Additionally, the first author is an Indigenous Latino from working class background and the second author is white from a middle-class background. Despite this, we know we occupied a privileged position doing this work and attempted to be as sympathetic and helpful as possible. This included providing a list of local resources along with the compensation our participants received. Additionally, we had multiple organizations who had previously agreed to provide services if a crisis emerged. Throughout the analysis, we strived to represent women's narratives and ways of knowing, conscious of our privilege and with an unwavering commitment to their voices. Additionally, we shared our professional experience as well as information about our lives. Answering any questions participants had about us, our university affiliation, or the goals of our research.

### Benefits and Risks of Study Participation

This study was conducted during a particularly difficult time for research, during the early stages of the COVID-19 pandemic. As such, there are multiple potential benefits and risks for the Indigenous women who participated in this study. To mitigate potential risks interviews were conducted remotely in order to product participant and project staff health and reduce any potential for COVID-19 exposure. An additional benefit of this approach was that participants could more easily schedule interviews around their schedules as well as it mitigated the need to arrange transportation to an interview location. A primary benefit being the potential therapeutic nature of having a venue to express and talk about one's experiences during times of high stress such as during tumultuous time of COVID-19. Alternatively, this same potential benefit may be a potential risk for participants if discussing the experience surrounding COVID-19 were to trigger unpleasant or stressful thoughts.

## Results

We describe the major themes that appeared in our research below in three small sections. The first addresses the negative effects COVID-19 had on the women we interviewed. The second deals with our participants and their inability to access health care during this time. Finally, we discuss how extended lockdowns and this pandemic resulted in women's increase use of drugs, alcohol, and tobacco.

### Mental Health

Out of the thirteen Indigenous women we spoke to, one of the most prominent themes that emerged was a general decline in individuals' mental health. Almost all the respondents we spoke with discussed how the COVID-19 pandemic, regional lockdowns, and the closure of schools and services negatively affected their mental health. This was exacerbated by women's gendered responsibilities like providing caretaker responsibilities and helping children with online education. Additionally, the women we spoke to discuss the multiple economic challenges associated with this pandemic and how it exacerbated their mental health. Two respondents said the following:

*I think the mental part is that is a big factor. [I got laid off] and there's been months where it's like, oh, my God, my rent, you know, my rent's expensive, I pay, you know, quite a bit amount of money on rent, right? And then it's like, “What do I do? Do I sacrifice my rent? Because we're going to go on another shutdown again, I need to buy groceries.”… and then my son's like, I just wish this COVID would go away. You know, my daughter, you know, 16 wants to get out and about but can't get out and about… they're both isolated. And some days are rougher and tougher in school, you know what I mean? And, it's hard to watch my son trying to do his, excuse me, try to do his work. And I don't have the proper skills to teach him how to tell time, how to do division and multiplication and stuff like that, right? So, I try my best*. (Jennine)

*Everything's really scary. My anxiety level is sky-high every time I go out or anything. I already had issues with my anxiety*. (Laura)

Jennine and Laura summarize the multiple challenges they have experienced during COVID-19. Jennine specially describes how these challenges have created additional stress in her life, resulting in her declining mental health. Her status as a single mother also exacerbated these challenges. While previously she could access support from her family, the COVID-19 pandemic has prevented this type of contact. This has resulted in her taking on the responsibility of homeschooling her children, providing meals, and engaging in other household responsibilities. She noted how the additional responsibilities and the inability to rest, was severely affecting her mental health. The recent loss of her job also exacerbated her mental health decline. Laura describes how this pandemic has exacerbated her existing anxiety issues which are severely triggered when leaving her home which presents the elevated risk of contracting this virus. Respondents also describe existing mental health issues like anxiety and post-traumatic stress disorder becoming worse during multiple lockdowns. Jennine, Laura and the experiences of other women in our study are consistent with other work dealing with Indigenous peoples. This small body of research demonstrates that Indigenous communities more so than other populations were adversely affected by the lockdown measures, physical distancing, and the pandemic as a whole (Statistics Canada, 2020).

There is evidence in the existing literature base that the qualitative findings on the impact of COVID-19 on mental health are not limited to the current study population. One cross-sectional study in Canada indicated an increased mental health concern burden among Indigenous participants surveyed, compared to white or Asian study participants (Lawal et al., 2021). Previous research has indicated that financial stresses and “food worry” exacerbate COVID-19 related health concerns among Canadians; food worry was associated increased odds of participants feeling anxious or worried as well as increased suicidal thoughts, even after controlling for other factors (McAuliffe et al., 2021). Taken together there is increasing evidence that the COVID-19 pandemic has and is continuing to impact the mental health and wellbeing of persons across the globe, with Indigenous populations being disproportionately vulnerable due to historical and structural inequities.

### Access to Health Care

The women we spoke to mentioned having a difficult time accessing health care during the pandemic. This included difficulty physically going to healthcare providers and issues accessing medical care remotely. For example, our respondents mentioned difficulty using online-based medical care. Our respondents mentioned feeling awkward or lost during these phone appointment conversations.

*And then you know, I mean, it was only a five-minute call. I find my doctors a little bit dimwitted and a bit rushed. So, you know… You're on the phone. You're trying to think. I booked an appointment with my doctor, I booked it in September. And my virtual interview or whatever interview over the phone was a month later*. (Jenny)

*It's hard to go to the doctors and stuff like that. So, I'm- sometimes when I even have to go and see my doctor it's hard for me to get down there*. (Alice)

*I have taken 2 COVID tests… so far. But that's just to make sure that I'm safe…did go to… the Aboriginal Bus that kind parked somewhere, and if you need a COVID test, you can just go there and you don't have to make an appointment and wait a couple of days. You just gotta find the bus, where it's that day*. (Jennifer).

Both Jenny and Asley discuss the challenges they had accessing healthcare. Jenny mentioned the general challenges of using remote health services. She felt uneasy using these services, and it seemed as if the physician was rushed and, in a hurry, to end the call. Alice was unable to access medical care given their limited access to technology. So, the only option was to access services in person. However, at the start of the COVID-19 pandemic, Alice could not receive medical attention in person. In her case, she had bronchitis which made it difficult to wear a mask. With masks restrictions in Toronto and no access to a medical note, she has been home bound for approximately a year. The inability to gain medical care also extended to accessing mental health services. Jennifer was able to access medical services but only by relying on an Indigenous based mobile van. Taking this approach, she was able to speak to someone in person and without needing an appointment. However, she first needed to find this bus which she did via social media or by contacting her networks. Women unfamiliar with these services faced similar changes to those of Jen and Alice. These findings are even more jarring given the widely accessible health care system in Canada.

Changing access to healthcare due to COVID-19, including difficulty seeing a doctor in person and increases in telemedicine may prove problematic in a variety of ways, for example, one commentary in the Journal of Substance Abuse Treatment indicated a series of challenges including increased prescription flexibility, which at times helpful, might contribute to the illicit and harmful use of certain controlled substances such as opioids (Wendt et al., 2021). Further, there is evidence of high prevalence of mental health concerns (moderate- to high depression, anxiety, stress, and low levels of wellbeing) among Indigenous populations in Bangladesh (Faruk et al., 2021). It is possible that similar results are to be found in Canada and globally.

### COVID-19 and Increased Substance Use

Our respondents discussed an increase in the consumption of alcoholic beverages. This was directly tied to the COVID-19 pandemic, lockdown measures and spending extended periods indoors. Ciara mentioned the following: “I just started drinking really heavily. I drink about a 12 pack a day now.” When we asked Jenny about how COVID-19 has affected her drinking, she said, “I think it was excessive.” For multiple respondents, their drinking became so acute that they began having problems paying for everyday living expenses like food and rent. With the general lack of medical, mental health and rehabilitation treatment, these problems have gone unchecked.

The people we spoke to also began to use drugs at higher rates than before. Most respondents began to use cannabis more frequently compared to other drugs. However, some respondents reported using other drugs like pharmaceutical pills and heroin. When we asked if her drug use had increased, Alice said the following: “*Yes, yes, yes. I smoke marijuana a lot now.” (all drug use) Yeah, it's definitely gone up. Two other respondents said shared this during an interview:*

*Well at the beginning of the pandemic, or whatever I kind of hampered down. Like a couple of years ago, I've never been into weed or marijuana or whatever you wanna call it, it was never something typically and I was never into drugs my whole life…And at the beginning of this whole thing, there was nothing to do and there were edibles and I could smoke. Whereas, in the past I've used it from my insomnia*. (Robin, Age-N/A)

*I smoke marijuana and I feel like I smoke more of everything since the start (of covid)*. (Gina)

The respondents included in this study shared similar sentiments to Robin, Alice and Gina. Most noted an increase in the use of drugs, with marijuana being the most widely used. However, the individuals we spoke to also reported using drugs in combination with other substances like alcohol. The increase was directly tied to COVID-19 and lockdown measures in their respective region.

Finally, our respondents also mentioned an increased use of tobacco products. This mostly included prepackaged cigarettes. With the lack of social interaction and the increased monotony of staying home, the individuals we spoke to began to fill their time with smoking on a more regular basis.

*Yeah, it's definitely gone up, definitely. I noticed especially at the beginning of the pandemic I was smoking – I don't usually smoke at work, and I was finding myself going on break at work and buying a pack of cigarettes*. (Patty)

Alice shared a similar sentiment:

…*all the money that I get [goes] to my drinking and my weed smoking and my cigarettes take up all that money. It's mostly food. Like I don't know where to go get food*.

Most of our respondents discussed an increase in the use of tobacco products. While some used vapor pens or e-cigarettes, most smoked traditional cigarettes. Although this initial increase began due to stay-at-home orders, it continued as the pandemic progressed. Given the already precarious financial status of many of our respondents, they often began to experience economic hardships due to their increased tobacco and substance consumption. While the existing evidence in Canada is limited, there is This troubling finding is in concordance with a study from the United Kingdom (U.K.), which documented not only increases in alcohol consumption from before the pandemic among study participants, but also a statistically significant relationship between alcohol use and mental health (Jacob et al., 2021).

## Discussion

In agreement with the limited existing scientific data and anecdotal data, our qualitative research suggests that COVID-19 has had a profound impact on the Indigenous women interviewed in terms of stress levels, mental health, and wellbeing, as well as corresponding increases in their substance use. There is evidence of increased mental health concerns due to COVID-19 across the globe (Dong and Bouey, 2020; Fiorillo and Gorwood, 2020; Passos et al., 2020; Sheridan Rains et al., 2021). Further, there is emerging evidence that some women may be at increased risk for adverse mental health concerns related to COVID-19; particularly in relationship with maternal health, pregnancy and increased domestic violence concerns (Almeida et al., 2020; Ayaz et al., 2020; Salehi et al., 2020; Sediri et al., 2020; Sharma et al., 2020). Further, a letter to the editor addresses the disparate need to address increased mental health concerns among Indigenous populations globally due to the impact of the COVID-19 on already marginalized populations (Júnior et al., 2020). This, coupled with a study in Canada indicating increased mental health concerns associated with the pandemic among Indigenous populations compared to white and Asian populations (Lawal et al., 2021) indicates an increased need for both research and prevention efforts.

According to respondents, COVID-19 related Mental Health concerns were exacerbated by stressors related to school closures and economic concerns associated with the pandemic. In addition, some respondents (10) reported losing their jobs or resorting to being self-employed, adding to the stress of an already stressful time. This had a particularly strong impact for women participants, given gendered norms and expectations of child-rearing, leaving one respondent feeling that she must choose between her children's education and paying rent, as well as helping to manage her children's stress and mental health concerns regarding the pandemic.

Further, contributing to stress and economic concerns, study respondents reported having difficulty accessing health care services (including much needed mental health services), despite much of medical care going online during the pandemic. Further, pre-existing health concerns limited some participants' ability to participate in in-person care even when it was available due to mask restrictions and inability to breathe.

There is evidence that there is a lack of sufficient mental health services to address reported increased mental health concerns among Indigenous populations due to the COVID-19 pandemic (Júnior et al., 2020), increased resources are needed to combat this important public health concern. There is a continued call to leverage the COVID-19 pandemic to decolonize and improve Indigenous health in Canada and globally (Júnior et al., 2020; Richardson and Crawford, 2020).

Regarding substance use our findings were in agreement the limited available literature demonstrating an increase in substance use during the pandemic (Jacob et al., 2021), the female participants indicated increases in commercial tobacco, marijuana and alcohol consumption, even among those who either infrequently or did not use substances before the COVID-19 pandemic. These observations are in line with widely held theories on stress, coping and substance use.

A great strength of this study is that we utilized existing community relationships to quickly identify some critical COVID-19 related stress, mental health and increased substance use issues among Indigenous populations in the Greater Toronto Area. This study is not without limitations. Our small sample size (*N* = 13) and the qualitative nature of the project means that we are unable to generalize to wider Indigenous populations in the Greater Toronto Area or the rest of Canada. However, this qualitative study gives us preliminary evidence to move forward with future research and partnerships with Indigenous peoples to better understand the current and future impacts of COVID-19 on the mental health and wellbeing of Indigenous populations. Future mixed-methods work is needed to confirm generalizability and better understand these concerns both qualitatively and quantitatively.

Documenting health equity issues is one of the first steps in addressing health disparities. The current study has important implications for future research and policy surrounding COVID-19 and Indigenous health in Canada. It points to a need for increased resources targeted toward Indigenous populations. In particular, the study's findings document a need for not increased culturally appropriate mental health and financial resources, and funding of said resources, targeted to meet the needs of Indigenous populations in Canada. Community leaders, public health professionals, government officials and advocates can use study findings to better address social and policy gaps surrounding COVID-19, mental health and underlying risk factors for Indigenous communities in Canada and globally.

## Data Availability Statement

The datasets presented in this article are not readily available because participants did not give permission for the data to be public. Requests to access the datasets should be directed to the corresponding author.

## Ethics Statement

The studies involving human participants were reviewed and approved by University of Toronto Research Ethics Board (RIS Human Protocol Number: 36345). The patients/participants provided their written informed consent to participate in this study.

## Author Contributions

All authors listed have made a substantial, direct, and intellectual contribution to the work and approved it for publication.

## Funding

This study was supported by a Connaught Early Career Research grant.

## Conflict of Interest

The authors declare that the research was conducted in the absence of any commercial or financial relationships that could be construed as a potential conflict of interest.

## Publisher's Note

All claims expressed in this article are solely those of the authors and do not necessarily represent those of their affiliated organizations, or those of the publisher, the editors and the reviewers. Any product that may be evaluated in this article, or claim that may be made by its manufacturer, is not guaranteed or endorsed by the publisher.

## Footnotes

^1^While we prefer doing interviews and fieldwork in person. Doing virtual fieldwork was not particularly challenging. We believe this was the case given our existing relationships with women we interviewed.

^2^All of the women we interviewed self-identified as Indigenous. For the parts of this chart were information is not available “N/A” this was due to respondents not sharing this specific information for personal reasons, reasons related to anonymity or they simply did not want to share this information.
